# Treinamento de Caminhada Melhora a Variabilidade da Pressão Arterial Ambulatorial em Claudicantes

**DOI:** 10.36660/abc.20190822

**Published:** 2021-05-06

**Authors:** Marcel da Rocha Chehuen, Gabriel Grizzo Cucato, Celso Ricardo Fernandes de Carvalho, Antonio Eduardo Zerati, Anthony Leicht, Nelson Wolosker, Raphael Mendes Ritti-Dias, Claudia Lucia de Moraes Forjaz

**Affiliations:** 1 Escola de Educação Física e Esporte Universidade de São Paulo São PauloSP Brasil Universidade de São Paulo - Escola de Educação Física e Esporte, São Paulo, SP - Brasil; 2 Northumbria University Newcastle Upon Tyne Reino Unido Northumbria University, Newcastle Upon Tyne, Reino Unido; 3 Universidade de Sao Paulo Faculdade de Medicina Hospital das Clinicas (HCFMUSP) São PauloSP Brasil Hospital das Clinicas (HCFMUSP), Faculdade de Medicina, Universidade de Sao Paulo, São Paulo, SP - Brasil; 4 James Cook University Queensland Austrália James Cook University, Queensland - Austrália; 5 Hospital Israelita Albert Einstein São PauloSP Brasil Hospital Israelita Albert Einstein, São Paulo, SP - Brasil; 6 Universidade Nove de Julho São PauloSP Brasil Universidade Nove de Julho - Programa de Pós-Graduação em Ciências da Reabilitação, São Paulo, SP - Brasil

**Keywords:** Claudicação Intermitente, Caminhada, Pressão Arterial, Monitoração Ambulatorial da Pressão Arterial, Fraqueza Muscular, Treinamento Aeróbico

## Abstract

**Fundamento:**

O treinamento de caminhada (TC) melhora a capacidade de caminhar e reduz a pressão arterial (PA) clínica em pacientes com doença arterial periférica (DAP), mas seus efeitos na PA ambulatorial permanecem desconhecidos.

**Objetivo:**

Investigar o efeito de 12 semanas de TC na PA ambulatorial e sua variabilidade em pacientes com DAP.

**Métodos:**

Trinta e cinco pacientes do sexo masculino com DAP e sintomas de claudicação foram alocados aleatoriamente em dois grupos: controle (n = 16, 30 min de alongamento) e TC (n = 19, 15 séries de 2 minutos de caminhada na frequência cardíaca em que ocorreu limiar de dor intercalados por 2 minutos de repouso em pé). Antes e depois de 12 semanas, a PA ambulatorial de 24 horas foi avaliada. Os índices de variabilidade da PA ambulatorial avaliados em ambos os momentos incluíram o desvio-padrão de 24 horas (DP_24_), o desvio-padrão ponderado de vigília e sono (DP_vs_) e a variabilidade real média de 24 horas (VRM_24_). Os dados foram analisados por ANOVAs mistas de dois fatores, considerando significativo P<0,05.

**Resultados:**

Após 12 semanas, nenhum dos grupos apresentou alterações na PA de 24 horas, vigília e sono. O TC diminuiu as variabilidades da PA sistólica e média (PA sistólica – 13,3 ± 2,8 vs 11,8 ± 2,3; 12,1 ± 2,84 vs 10,7 ± 2,5; e 9,4 ± 2,3 vs 8,8 ± 2,2 mmHg; PA média – 11,0 ± 1,7 vs 10,4 ± 1,9; 10,1 ± 1,6 vs 9,1 ± 1,7; e 8,0 ± 1,7 vs 7,2 ± 1,5 mmHg para DP_24_, DPvs e VRM_24_, respectivamente). Nenhum dos grupos apresentou mudanças significantesnos índices de variabilidade da PA diastólica após 12 semanas.

**Conclusões:**

O TC não altera os níveis ambulatoriais da PA, mas diminui a sua variabilidade em pacientes com DAP. Essa melhora pode ter um impacto favorável no risco cardiovascular de pacientes com DAP sintomática. (Arq Bras Cardiol. 2021; 116(5):898-905)

## Introdução

A claudicação intermitente, o sintoma mais prevalente da doença arterial periférica (DAP), prejudica a capacidade de locomoção, afetando os níveis de atividade física^1^ e a qualidade de vida do paciente.^2^ Além disso, essa limitação funcional está associada ao aumento das taxas de eventos cardiovasculares fatais e não fatais nessa população.^3^

Dentre as doenças cardiovasculares, a hipertensão arterial é uma comorbidade frequente, afetando mais de 80% dos pacientes com DAP,^4^ Pacientes com DAP comumente apresentam valores de pressão arterial (PA) clínica e, principalmente, ambulatorial mais altos que indivíduos saudáveis.^5^ Recentemente a capacidade de caminhada foi negativamente associada com a PA ambulatorial na DAP,^6^ indicando um pior controle pressórico em pacientes com maior comprometimento funcional. Assim, estratégias terapêuticas que aumentem a capacidade funcional, como o treinamento de caminhada, podem melhorar os desfechos cardiovasculares e reduzir o risco cardiovascular nesse grupo.

Recentemente, demonstramos que o treinamento de caminhada (TC) supervisionado melhora a capacidade de caminhada e reduz a PA clínica de pacientes com DAP sintomática,^7^ porém seus efeitos na PA ambulatorial permanecem desconhecidos. Essa é uma questão importante, uma vez que a PA ambulatorial é um preditor mais forte de mortalidade por causas cardiovasculares do que a PA clínica.^8^ Além disso, um estudo anterior não relatou nenhum efeito do treinamento resistido de nos níveis ambulatoriais da PA, mas observou uma melhora na variabilidade da PA ambulatorial, que é um forte marcador de lesão de órgãos-alvo, eventos cardiovasculares e mortalidade.^10^ Uma vez que o treinamento aeróbico, como a caminhada, promove redução considerável nos níveis de PA ambulatorial em comparação ao treinamento resistido em populações normotensas e hipertensas,^11^ é possível supor que esta modalidade de exercício também possa melhorar a PA ambulatorial e sua variabilidade em pacientes com DAP, o que precisa ser verificado. Assim, o objetivo deste estudo foi investigar os efeitos do TC na PA ambulatorial e sua variabilidade em pacientes com DAP sintomática.

## Métodos

### População do estudo

Este é um dado complementar de um estudo anterior.^7^ Os pacientes foram recrutados no Ambulatorio de Cirurgia Vascular do Hospital das Clínicas da Universidade de São Paulo, Brasil. Foram convidados pacientes do sexo masculino com diagnóstico prévio de DAP e sintomas de claudicação intermitente.^12^ Os critérios de inclusão foram: (a) idade ≥ 50 anos; (b) índice tornozelo-braquial (ITB) ≤ 0,90^11^^,^^12^; c) DAP classe II segundo os critérios de Fontaine;^13^ (d) índice de massa corporal <35 kg/m^2^; (e) PA sistólica em repouso <160 mmHg e PA diastólica <105 mmHg; (f) não estar tomando β-bloqueadores ou bloqueadores dos canais de cálcio não diidropiridínicos; (g) ausência de neuropatia autonômica cardiovascular nos pacientes diabéticos;^14^ (h) capacidade de caminhar por pelo menos 2 minutos a 3,2 km/h em uma esteira; (i) capacidade de realizar um teste incremental em esteira que seja limitado por por sintomas de claudicação intermitente; (j) ausência de isquemia miocárdica ou arritmias complexas durante um teste máximo em esteira; (k) diminuição de pelo menos 15% no ITB após um teste máximo em esteira; e (l) não estar envolvido em programa de exercícios físicos. Além disso, os pacientes não eram incluídos se tivessem pelo menos um dos seguintes critérios: 1) cirurgia de revascularização ou angioplastia há menos de um ano; 2) uso de vasodilatadores periféricos, 3) amputação de membros inferiores e 4) problemas ortopédicos que contraindicassem o TC. Os indivíduos foram excluídos quando seus medicamentos foram alterados durante o estudo. O protocolo do estudo foi registrado no Brazilian Clinical Trials (RBR-7M3D8W) e aprovado pelo Comitê de Ética em Pesquisa em Seres Humanos da Escola de Educação Física e Esporte da Universidade de São Paulo (processo: 39-2008/55) e do Hospital das Clínicas (processo: 1179/09), sendo conduzido de acordo com a Declaração de Helsinque. Um termo de consentimento livre e informado foi obtido de todos os pacientes antes da participação.

### Triagem dos participantes

O diagnóstico de DAP foi feito com base no histórico clínico e na medida do ITB em repouso e após teste máximo em esteira.^15^ A PA sistólica do braço foi medida pelo método auscultatório e a PA sistólica do tornozelo de cada perna foi avaliada com um doppler vascular (Martec, DV 6000, Ribeirão Preto, Brasil). Para cada paciente, foi registrado o ITB de menor valor. A massa corporal e a altura foram medidas (Welmy, 110, São Paulo, Brasil), e o índice de massa corporal foi calculado. A PA braquial em repouso foi medida em duas visitas, sendo o valor médio calculado e utilizado na análise. Em cada visita, após cinco minutos em repouso sentado, foram realizadas três medidas auscultatórias da PA em cada braço, sendo registrado o maior valor médio. O uso de medicamentos e os hábitos de exercícios foram avaliados por meio de entrevista, Nos diabéticos, a presença de neuropatia autonômica cardiovascular foi avaliada de acordo com as recomendações da American Diabetes Association.^14^ O tratamento medicamentoso foi mantido constante para todos os pacientes ao longo do estudo.

### Desenho do estudo

**Figura 1:**
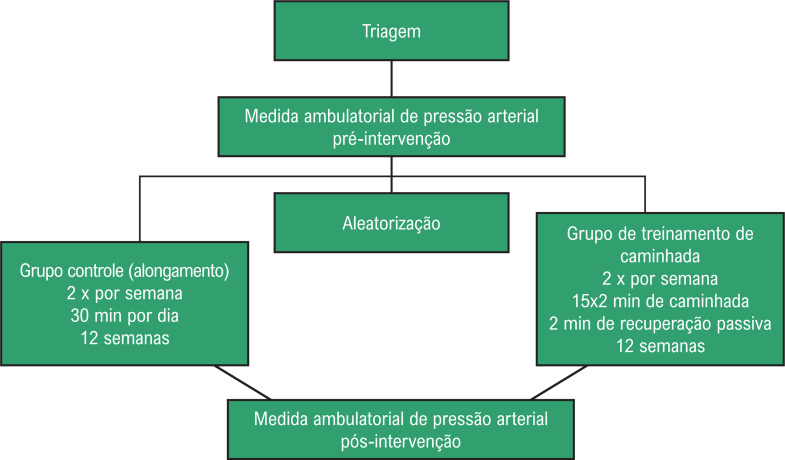
Desenho experimental do estudo.

O desenho experimental está descrito na Figura 1. O estudo foi composto por uma triagem inicial que incluiu um teste máximo em esteira utilizando o protocolo de Gardner para avaliar o limiar de dor.^16^ Em seguida, os indivíduos que atenderam a todos os critérios do estudo realizaram uma monitorização ambulatorial da PA de 24 horas no início e 12 semanas após a intervenção. Os pacientes foram alocados de forma aleatória, por meio de um programa online específico (www.randomizer.org) em dois grupos: grupo treinamento de caminhada (GTC) e grupo controle (GC).

Para todas as avaliações, as recomendações prévias incluíam a não realização de exercício vigoroso nas 48 horas anteriores, realização de uma refeição leve 2 horas antes, a não ingestão de alimentos com propriedades estimulantes como cafeína, bebida alcoólica ou o consumo de tabaco nas 12 horas anteriores. As avaliações clínicas foram realizadas pela manhã em um laboratório com temperatura controlada (20-22 °C).

## Medidas

### Desfecho primário: pressão arterial ambulatorial

A monitorização ambulatorial da PA foi realizada com um aparelho oscilométrico não invasivo (SpaceLabs Medical Inc, 90207, Washington, EUA) posicionado no braço não dominante e programado para realizar medidas a cada 15 minutos durante 24 horas. A precisão do dispositivo foi confirmada por um esfigmomanômetro de mercúrio antes do uso.

Para a análise, os níveis ambulatoriais de PA sistólica, diastólica e média foram calculados pela média de todas as medidas realizadas por 24 horas, bem como durante os períodos de vigília e sono relatados pelo paciente. Além disso, a variabilidade da PA ambulatorial foi calculada para a PA sistólica, diastólica e média usando três índices diferentes:^17^ desvio-padrão de 24 horas (DP_24_); desvio-padrão ponderado em vigília e durante o sono (DP_VS_) e a variabilidade real média de 24 horas (VRM_24_). Esses índices foram calculados conforme relatado anteriormente.^17^ Resumidamente, o DP_24_ foi calculado pelo desvio-padrão (DP) ao longo de 24 horas ponderado pelo intervalo de tempo entre as medidas. DPvs foi calculado pela média de DP de vigília e sono corrigido para o número de horas de cada um desses períodos [ou seja, DPvs = [(DP vigília x horas de vigília) + (DP sono x horas de sono)] / horas de vigília + horas de sono]. A VRM_24_ foi calculada pela média das diferenças absolutas entre as medições consecutivas, contabilizando a ordem de medição pela seguinte fórmula:

$$VRM_{24}=\frac{1}{\sum {}_{w}}\sum_{k=1}^{n-1}w\times \left|PA_{k+1}-PA_{k}\right|$$

Onde k varia de 1 a N−1, PA o valor da pressão arterial e w é o intervalo de tempo entre PAk e PAk+1. N é o número de registros válidos da PA.

### Intervenções

Detalhes das intervenções foram relatados anteriormente.^7^ Resumidamente, as intervenções foram realizadas duas vezes por semana durante 12 semanas e supervisionadas por um dos pesquisadores. Os pacientes do GC realizaram exercícios de alongamento por 30 minutos. Os pacientes do GTC realizaram 15 séries de 2 minutos de caminhada em uma esteira, intercaladas por 2 minutos de repouso. Durante cada série de caminhada, a velocidade foi mantida em 3,2 km/h e a intensidade foi ajustada pela inclinação da esteira para manter a frequência cardíaca dentro de 4 bpm da frequência cardíaca obtida no limiar de dor avaliado durante o teste máximo em esteira^18^ (por exemplo, se o paciente relatou o limiar de dor durante teste máximo em esteira a 100 bpm, a frequência cardíaca durante cada série era mantida entre 96 a 104 bpm).

### Análise estatística

Conforme descrito anteriormente,^7^ o tamanho da amostra foi estimado considerando-se um poder de 90%, erro alfa de 5% e desvio-padrão de 3 mmHg para a PA sistólica. O tamanho mínimo necessário para detectar uma diferença de 4 mmHg foi de 7 indivíduos em cada grupo.

A normalidade da distribuição dos dados e a homogeneidade da variância foram avaliadas pelos testes de Shapiro-Wilk e Levene, respectivamente. Distribuições que não atenderam aos critérios de normalidade foram normalizadas usando transformações logarítmicas. No início do estudo, as diferenças entre os grupos foram identificadas por meio do teste qui-quadrado (comorbidades foi prevalência da terapia medicamentosa) ou teste t de Student não pareado (variáveis contínuas). Os efeitos das intervenções foram avaliados pela ANOVA mista de dois fatores (Statsoft, Statistic for Windows 4.3, Oklahoma, EUA), tendo como fatores grupo (GC e GTC) e fase do estudo (início e 12 semanas). Teste post-hoc de Newman-Keuls foi usado quando necessário. O valor de p<0,05 foi considerado significante e os dados foram apresentados em média ± DP.

## Resultados

**Figura 2:**
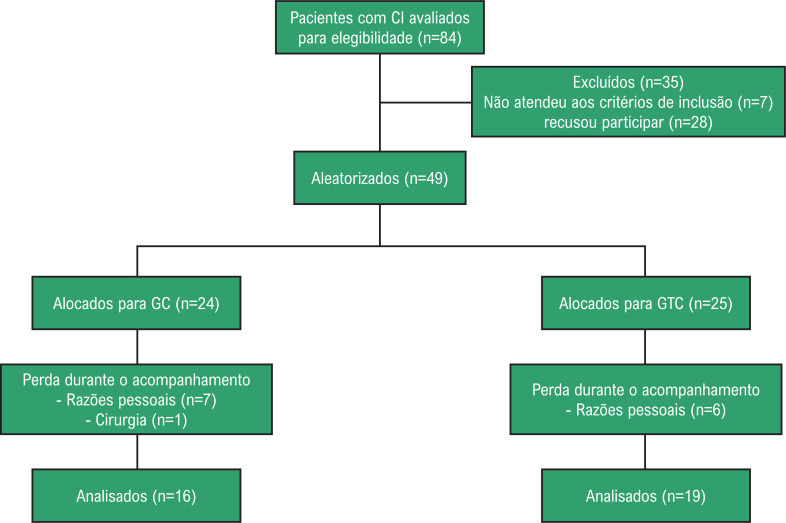
Fluxograma do estudo CI: claudicação intermitente, GC: Grupo controle, GTC: Grupo treinamento de caminhada

O fluxograma de pacientes está representado na Figura 2. Oitenta e quatro pacientes foram triados, mas 35 foram excluídos por não atenderem aos critérios de elegibilidade (n = 7) ou não aceitaram participar (n = 28, falta de disponibilidade para realização dos treinamento). Os 49 pacientes restantes foram alocados aleatoriamente no GC (n = 24) e no GTC (n = 25). Quatorze pacientes desistiram por circunstâncias não relacionadas ao estudo, de forma que a amostra final foi composta por 35 pacientes (GC, n = 16; GTC, n = 19).

Esses grupos tinham características iniciais semelhantes quanto à idade, grau de obesidade, níveis clínicos de PA, limitações da doença, comorbidades e uso de medicamentos (Tabela 1).

**Tabela 1:** Características dos pacientes alocados nos grupos controle e treinamento de caminhada

	GC (n = 16)	GTC (n = 19)	Valor p
Idade (anos)	62 ± 7	63 ± 7	0,64
Índice de massa corporal (kg/m^2^)	25,7 ± 3,9	26,1 ± 3,1	0,76
Índice tornozelo-braquial	0,60 ± 0,12	0,62 ± 0,14	0,61
Distância de início de claudicação (m)	319 ± 152	277 ± 164	0,45
Distância total caminhada (m)	759 ± 305	624 ± 255	0,16
PA sistólica clínica (mmHg)	136 ± 19	133 ± 14	0,60
PA diastólica clínica (mmHg)	79 ± 10	77 ± 9	0,53
**Comorbidades**			
Obesidade (%)	12,5	10,5	0,55
Hipertensão (%)	81,3	84,2	0,89
Diabetes Mellitus (%)	25,0	21,1	0,61
Dislipidemia (%)	100,0	89,5	0,17
Fumantes atuais (%)	37,5	26,3	0,38
Doença cardíaca/acidente vascular cerebral (%)	18,8	21,1	0,80
**Terapia medicamentosa**			
Aspirina (%)	93,8	100,0	0,28
Estatina (%)	62,5	78,9	0,83
Inibidor da enzima de conversão da angiotensina (%)	43,8	68,4	0,20
Diuréticos (%)	25,0	47,4	0,17
Bloqueador do canal de cálcio (%)	18,8		0,86
Hipoglicemiante oral (%)	18,8		0,69
**Número de anti-hipertensivos**			
Monoterapia	50,0		0,76

*Dados apresentados em média* ± *DP ou porcentagem (%). PA: pressão arterial. Variável contínua* – *teste t de Student não pareado. Variável categórica* – *teste do qui-quadrado. CG - Grupo controle, GTC - Grupo treinamento de caminhada*.

Os níveis de PA ambulatorial foram semelhantes entre os grupos no início do estudo, e nenhum deles apresentou qualquer mudança significante na PA de 24 horas, vigília e sono após as 12 semanas de intervenção (Tabela 2).

**Tabela 2:** Níveis de pressão arterial ambulatorial medidos no início e após o período de intervenção de 12 semanas no grupo treinamento de caminhada e o grupo controle

	GC (n = 16)		GTC (n = 19)		P grupo	P fase do estudo	P interação
	início	12 semanas	início	12 semanas			
**24h**							
PA sistólica (mmHg)	130 ± 14	132 ± 15	128 ± 14	126 ± 11	0,51	0,74	0,21
PA diastólica (mmHg)	78 ± 7	80 ± 7	78 ± 12	76 ± 10	0,44	0,42	0,16
PA média (mmHg)	96 ± 9	98 ± 8	94 ± 9	93 ± 9	0,32	0,60	0,14
**Vigília**							
PA sistólica (mmHg)	135 ± 14	137 ± 16	130 ± 14	129 ± 12	0,16	0,74	0,44
PA diastólica (mmHg)	83 ± 7	84 ± 7	80 ± 12	79 ± 11	0,16	0,41	0,35
PA média (mmHg)	101 ± 9	103 ± 9	96 ± 10	95 ± 10	0,08	0,60	0,25
**Sono**							
PA sistólica (mmHg)	119 ± 16	121 ± 16	124 ± 16	122 ± 12	0,50	0,85	0,51
PA diastólica (mmHg)	69 ± 9	71 ± 8	73 ± 9	71 ± 11	0,61	0,80	0,32
PA média (mmHg)	87 ± 11	89 ± 11	89 ± 9	89 ± 9	0,63	0,82	0,33

*Dados apresentados em média* ± *desvio padrão. PA: pressão arterial. ANOVA de dois fatores (grupo e fase do estudo). CG: Grupo controle, GTC: Grupo treinamento de caminhada*.

Os índices de variabilidade da PA avaliados no início do estudo foram semelhantes entre o GTC e o GC. Houve interação significante entre grupos e a fase de estudo para os índices de variabilidade da PA sistólica e média (todos P<0,05), mostrando redução do DP_24_, DP_vs_ e VRM_24_ da PA sistólica e média apenas no GTC (Tabela 3, Figura 3). Nenhum grupo teve qualquer mudança significante nos índices de variabilidade da PA diastólica.

**Tabela 3:** Índices de variabilidade da pressão arterial ambulatorial avaliados no início e após o período de intervenção de 12 semanas para os grupos treinamento de caminhada e controle

	GC (n = 16)		GTC (n = 19)		P grupo	P fase do estudo	P interação
	início	12 semanas	início	12 semanas			
**DP**_24_							
PA sistólica (mmHg)	14,6 ± 3,0	15,5 ± 3,9	13,3 ± 2,8	11,8 ± 2,3*#	0,01	0,65	0,04
PA diastólica (mmHg)	10,9 ± 1,8	11,2 ± 1,7	9,7 ± 2,3	10,0 ± 2,5	0,06	0,49	0,68
PA média (mmHg)	12,0 ± 2,6	13,0 ± 3,0	11,0 ± 1,7	10,4 ± 1,9#	0,01	0,71	0,04
**DP**_vs_							
PA sistólica (mmHg)	12,2 ± 2,4	12,7 ± 3,0	12,1 ± 2,4	10,7 ± 2,5*#	0,18	0,27	0,03
PA diastólica (mmHg)	8,7 ± 1,3	9,0 ± 1,6	9,0 ± 1,8	8,9 ± 2,2	0,98	0,95	0,48
PA média (mmHg)	10,0 ± 2,1	10,7 ± 2,2	10,1 ± 1,6	9,1 ± 1,7*##	0,23	0,82	0,01
**VRM**_24_							
PA sistólica (mmHg)	9,4 ± 2,1	10,7 ± 2,4*	9,4 ± 2,3	8,8 ± 2,2#	0,18	0,28	0,02
PA diastólica (mmHg)	6,9 ± 1,8	7,3 ± 1,8	7,3 ± 2,3	7,2 ± 1,6	0,75	0,67	0,54
PA média (mmHg)	8,1 ± 1,9	8,6 ± 1,7	8,0 ± 1,7	7,2 ± 1,5*#	0,15	0,88	0,01

*Valores apresentados em média* ± *desvio-padrão. DP_24_* = *desvio-padrão ponderado de 24 horas; DPvs* = *desvio-padrão ponderado em vigília e durante o sono; VRM* = *variabilidade real média. ANOVA de dois fatores (grupo e fase do estudo)*.

* *Diferente do início (p <0,05);*

# *Diferente do GC (p <0,05). GC: Grupo controle, GTC: Grupo treinamento de caminhada, PA: pressão arterial*.

**Figura 3:**
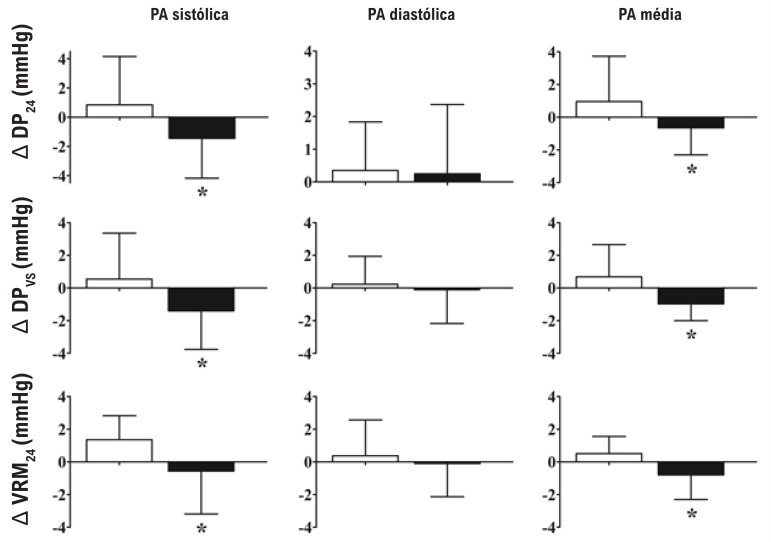
Alteração absoluta (Δ) da variabilidade da pressão arterial ambulatorial para o grupo controle (GC – barras brancas) e grupo de treinamento de caminhada (GTC – barras pretas). PA: pressão arterial; DP_24_, desvio-padrão acima de 24 horas ponderado pelo intervalo de tempo entre leituras consecutivas; DPvs, a média dos DPs diurnos e noturnos ponderados para a duração do intervalo diurno e noturno, VRM_24_: variabilidade real média ponderada para o intervalo de tempo entre leituras consecutivas de registros de PA ambulatorial de 24 horas. * p <0,05 vs. GC.

## Discussão

O principal achado deste estudo foi que 12 semanas de TC diminuíram os índices de variabilidade da PA sistólica e média, sem alterar os níveis de PA ambulatorial.

No presente estudo, 12 semanas de TC não alteraram a PA ambulatorial em pacientes com DAP, o que contrasta com estudos realizados com indivíduos normotensos e hipertensos^19^ que relataram consistentemente reduções em torno de 3 mmHg para PA sistólica e diastólica ambulatorial após treinamento aeróbico. No entanto, 12 semanas de treinamento resistido também não alteraram a PA ambulatorial em pacientes com DAP.^9^ Assim, tem-se a hipótese de que episódios frequentes de isquemia durante as atividades diárias dos pacientes com DAP produzem dor de claudicação, estresse oxidativo e acúmulo metabólico, aumentando a atividade do nervo simpático e, consequentemente, mitigando qualquer possível efeito do exercício físico sobre os níveis de PA ambulatorial.^20^ Outra possível explicação, entretanto, pode ser a duração muito curta do programa, uma vez que um estudo anterior^21^ realizado com hipertensos idosos não mostrou alteração dos níveis de PA ambulatoriais após 6 meses de treinamento, após 12 meses de intervenção.

Apesar da ausência de alteração nos níveis de PA ambulatorial, foram observadas reduções nas variabilidades sistólica e média da PA ambulatorial para todos os índices de variabilidade: DP_24_, DPvs e VRM_24_. Esses resultados estão de acordo com estudo anterior com treinamento resistido em pacientes sintomáticos com DAP.^9^ Esse resultado é coerente com a ideia de que mudanças no controle autonômico precedem alterações nos níveis da PA, uma vez que a variabilidade da PA reflete principalmente o controle autonômico da PA.^22^^,^^23^ Além disso, esses resultados também estão de acordo com nossos achados anteriores de uma melhora na modulação autonômica cardíaca e sensibilidade barorreflexa, marcadores de controle autonômico, após TC em pacientes com DAP.^7^ A ausência de alterações na variabilidade da PA ambulatorial diastólica também é coerente com a ausência de efeitos do treinamento de caminhada na resistência vascular da panturrilha, como descrito anteriormente.^7^

Mesmo sem quaisquer alterações nos níveis de PA ambulatorial, a diminuição da variabilidade da PA ambulatorial obtida com o TC pode ter implicações clínicas relevantes. A variabilidade da PA tem sido associada à presença e progressão de lesões de órgãos alvo, bem como à incidência de eventos cardiovasculares,^10^ levando a um pior prognóstico cardiovascular.^8^ Assim, a diminuição induzida pelo TC pode ter impacto favorável no risco cardiovascular de pacientes com DAP, reforçando a recomendação do TC para esses pacientes.

Este estudo apresenta algumas limitações que devem ser reconhecidas. Foi realizado apenas com homens e as adaptações induzidas pelo treinamento podem diferir entre os sexos.^24^^,^^25^ Assim, estudos futuros devem investigar o impacto do TC na PA ambulatorial e sua variabilidade também em mulheres, principalmente idosas, que podem apresentar maior risco cardiovascular do que os homens.^24^ O presente estudo também examinou apenas pacientes com sintomas de claudicação, e novos estudos devem examinar os efeitos do TC em outros grupos de pacientes, como os assintomáticos (estágio 1) e naqueles que apresentam diminuição dos níveis pressóricos ambulatoriais após o TC. Por fim, o programa durou 12 semanas, duração que melhora a capacidade funcional e os parâmetros clínicos cardiovasculares desses pacientes,^7^ mas um período de treinamento mais longo pode ser necessário para diminuir os níveis pressóricos ambulatoriais.

## Conclusão

Em conclusão, 12 semanas de TC diminui a variabilidade ambulatorial da PA em homens com DAP sintomática.
