# Supplementary material for: Treinamento de Caminhada Melhora a Variabilidade da Pressão Arterial Ambulatorial em Claudicantes
Source: Arq Bras Cardiol. 2021 May 6;116(5):898–905. [Article in Portuguese] doi: 10.36660/abc.20190822 (PMC8121473; doi:10.36660/abc.20190822)
Supplement: Translation

# Translation: Walking Training Improves Ambulatory Blood Pressure Variability in Claudication

**Authors:** Marcel da Rocha Chehuen, Gabriel Grizzo Cucato, Celso Ricardo Fernandes de Carvalho, Antonio Eduardo Zerati, Anthony Leicht, Nelson Wolosker, Raphael Mendes Ritti-Dias, Claudia Lucia de Moraes Forjaz

**Affiliations:** 1 São PauloSP Brasil Universidade de São Paulo - Escola de Educação Física e Esporte, São Paulo, SP - Brasil; 2 Newcastle Upon Tyne Reino Unido Northumbria University, Newcastle Upon Tyne, Reino Unido; 3 São PauloSP Brasil Hospital das Clinicas (HCFMUSP), Faculdade de Medicina, Universidade de Sao Paulo, São Paulo, SP - Brasil; 4 Queensland Austrália James Cook University, Queensland - Austrália; 5 São PauloSP Brasil Hospital Israelita Albert Einstein, São Paulo, SP - Brasil; 6 São PauloSP Brasil Universidade Nove de Julho - Programa de Pós-Graduação em Ciências da Reabilitação, São Paulo, SP - Brasil

**Keywords:** Intermittent Claudication, Walking, Blood Pressure, Blood Pressure Monitoring Ambulatory, Muscle Weakness, Endurance Training

## Abstract

**Background:**

Walking training (WT) improves walking capacity and reduces clinic blood pressure (BP) in patients with peripheral artery disease (PAD), but its effects on ambulatory BP remains unknown.

**Objective:**

To investigate the effect of 12 weeks of WT on ambulatory BP and its variability in patients with PAD.

**Methods:**

Thirty-five male patients with PAD and claudication symptoms were randomly allocated into two groups: control (n = 16, 30 min of stretching) and WT (n = 19, 15 bouts of 2 min of walking at the heart rate of leg pain threshold interspersed by 2 min of upright rest). Before and after 12 weeks, 24-hour ambulatory BP was assessed. Ambulatory BP variability indices assessed at both time points included the 24-hour standard deviation (SD_24_), the awake and asleep weighted standard deviation (SD_dn_), and the 24-hour average real variability (ARV_24_). Data were analyzed by mixed two-way ANOVAs, considering P<0.05 as significant.

**Results:**

After 12 weeks, neither group had significant changes in 24-hour, awake and sleep BPs. The WT decreased systolic and mean BP variabilities (Systolic BP – 13.3±2.8 vs 11.8±2.3, 12.1±2.84 vs 10.7±2.5 and 9.4±2.3 vs 8.8±2.2 mmHg); Mean BP – 11.0±1.7 vs 10.4±1.9, 10.1±1.6 vs 9.1±1.7 and 8.0.±1.7 vs 7.2±1.5 mmHg) for SD_24,_ SD_dn_ and ARV_24_, respectively). Neither group had significant changes in diastolic BP variabilities after 12 weeks.

**Conclusions:**

The WT does not change ambulatory BP levels but decreases ambulatory BP variability in patients with PAD. This improvement may have a favorable impact on the cardiovascular risk of patients with symptomatic PAD. (Arq Bras Cardiol. 2021; 116(5):898-905)

## Introduction

Intermittent claudication, the most prevalent symptom of peripheral artery disease (PAD), impairs walking capacity, impacting on patient’s physical activity levels^1^ and quality of life.^2^ In addition, this functional limitation is associated with increased rates of fatal and non-fatal cardiovascular events in this population.^3^

Among cardiovascular diseases, arterial hypertension is a common comorbidity that affects more than 80% of the patients with PAD,^4^ who present higher clinic and specially higher ambulatory BP levels compared with healthy individuals.^5^ Interestingly, we recently demonstrated that walking capacity was negatively associated with ambulatory BP in PAD,^6^ indicating a poorer BP control in patients with greater functional impairment. Thus, therapeutic strategies that increase functional capacity, such as walking training, may improve cardiovascular outcomes and reduce cardiovascular risk in this group.

We have recently demonstrated that supervised walking training (WT) improves walking capacity in addition to reducing clinic BP in patients with symptomatic PAD,^7^ however its effects on ambulatory BP remains unknown. This is a very important issue, since ambulatory BP is considered a stronger predictor of all-cause and cardiovascular mortality than clinic BP.^8^ Additionally, a previous study reported no effect of resistance training on ambulatory BP levels, but an improvement in ambulatory BP variability,^9^ a new and strong marker for target-organ damage, cardiovascular events, and mortality.^10^ Given that aerobic training such as walking promotes considerable reduction on ambulatory BP levels compared to resistance training in normotensive and hypertensive populations,^11^ one may suppose that this mode of exercise can also improve ambulatory BP and its variability in patients with PAD, which needs to be checked. Thus, the aim of this study was to investigate the effects of WT on ambulatory BP and its variability in patients with symptomatic PAD.

## Methods

### Study Population

This is a complementary data from a previous study.^7^ Patients were recruited from the Clinic Hospital’s Vascular Unit, University of Sao Paulo, Brazil. Male patients previously diagnosed with PAD and with symptoms of intermittent claudication were invited. Inclusion criteria were: (a) age ≥ 50 years; (b) ankle-brachial index (ABI) ≤ 0.90;^11^^,^^12^ c) Fontaine stage II of PAD;^13^ (d) body mass index ≤ 35 kg/m^2^; (e) resting systolic BP ≤ 160 mmHg and diastolic BP ≤ 105 mmHg; (f) not taking β-blockers or non-dihydropyridine calcium channel blockers; (g) absence of cardiovascular autonomic neuropathy for diabetic patients;^14^ (h) ability to walk for at least 2 minutes at 3.2 km/h on a treadmill; (i) ability to undertake an incremental treadmill test limited by symptoms of intermittent claudication; (j) absence of myocardial ischemia or complex arrhythmias during a maximal treadmill test; (k) decrease of at least 15% in ABI after a maximal treadmill test; and (l) not engaged in any exercise program. In addition, patients were not included if they met at least one of the following criteria: 1) revascularization surgery or angioplasty less than one year earlier; 2) use of peripheral vasodilators, 3) lower limb amputation, and 4) orthopedic problems that contraindicate walking exercise. Subjects were excluded if they had their medications changed during the study. The study’s protocol was registered at the Brazilian Clinical Trials (RBR-7M3D8W) and approved by the Human Research Ethics Committee of the School of Physical Education and Sport of the University of Sao Paulo (process: 39-2008/55) and the Clinic Hospital (process:1179/09), being conducted in accordance with the Declaration of Helsinki. A written informed consent was obtained from all patients prior to participation.

### Participant screening

Diagnosis of PAD was made based on clinical history and ABI measurement at rest and after a treadmill maximal test.^15^ Arm systolic BP was measured using the auscultatory method, and ankle systolic BP of each leg was assessed with a Doppler ultrasound (Martec, DV 6000, Ribeirão Preto, Brazil). For each patient, the lowest ABI was recorded. Body mass and height were measured (Welmy, 110, São Paulo, Brazil), and body mass index was calculated. Resting brachial BP was measured in two visits, and the mean value was calculated and used for analysis. In each visit, after five minutes of seated rest, three auscultatory measurements were taken in each arm, and the highest mean value was recorded. Medication use and exercise habits were assessed via interview. In diabetic patients, the presence of cardiovascular autonomic neuropathy, was assessed according to the recommendations of the American Diabetes Association.^14^ Drug treatment was kept constant for all patients throughout the study.

### Design

The experimental protocol is shown in Figure 1. The study had an initial pre-screening including a maximal treadmill test following Gardner’s protocol for assessing pain threshold.^16^ Then, subjects who met all the study criteria underwent 24-hour ambulatory BP monitoring at baseline and after 12-weeks of intervention. Patients were randomized using a specific online program (www.randomizer.org) into two groups: walking training (WTG) and control (CG).

**Figure 1:** Experimental design of the study.

For all the assessments, recommendations included no vigorous exercise in the previous 48 hours, a light meal 2 hours before, no ingestion of food with stimulant properties such as caffeine, no alcoholic beverages or smoking in the previous 12 hours. Clinic assessments were conducted in the morning in a temperature-controlled laboratory (20-22°C).

## Measurements

### Primary outcome: ambulatory blood pressure

Ambulatory BP monitoring was performed with a noninvasive oscillometric device (SpaceLabs Medical Inc, 90207, Washington, USA) placed on the non-dominant arm and programmed to perform measurements every 15 minutes for 24 hours. The accuracy of the device was confirmed by a mercury sphygmomanometer prior to use.

For the analysis, ambulatory systolic, diastolic and mean BP levels were calculated by the average of all BP measurements taken during the 24 hours as well as during the awake and asleep periods reported by the patient. In addition, ambulatory BP variability was calculated for systolic, diastolic and mean BP using three different indices:^17^ the 24-hour standard deviation (SD_24_); the awake and asleep weighted standard deviation (SD_dn_), and the 24-hour average real variability (ARV_24_). These indices were calculated as previously reported. Briefly, SD_24_ was calculated by the standard deviation (SD) over 24 hours weighted for the time interval between measures. SD_dn_ was calculated by the mean of awake and asleep SD corrected for the number of hours of each of these periods [i.e SD_dn_ = [(awake SD x awake hours) + (asleep SD x asleep hours)]/(wake + asleep hours)]. ARV_24_ was calculated by the average of absolute differences between consecutive measurements accounting for the order of measurement using following formula:

$$ARM=\frac{1}{\sum {}_{w}}\sum_{k=1}^{n-1}w\times \left|BP_{k+1}-BP_{k}\right|$$

where k ranges from 1 to N−1, BP is the blood pressure and w is the time interval between BPk and BPk+1. N is the number of blood pressure readings.

### Interventions

Details of the interventions have been previously reported.^7^ Briefly, interventions were conducted twice a week for 12 weeks and supervised by one of the researchers. CG patients performed stretching exercises for 30 minutes. WTG patients performed 15 bouts of 2-minute walking on a treadmill intersected by 2 minutes of resting. During each walking bouts, speed was kept at 3.2 km/h and intensity was adjusted by setting the treadmill grade to maintain heart rate within 4 bpm of the heart rate obtained at the pain threshold assessed during maximal treadmill test^18^ (e.g., if the patient reported the pain threshold during maximal treadmill test at 100 bpm, the heart rate during each training session was kept between 96 to 104 bpm).

### Statistical analysis

As previously described,^7^ the sample size was estimated considering a power of 90%, alpha error of 5%, and standard deviation of 3 mmHg for systolic BP. The minimal sample size necessary to detect a difference of 4 mmHg was 7 subjects in each group.

Normality of data distribution and homogeneity of variance were evaluated using the Shapiro-Wilk and Levene tests, respectively. Skewed distributions were normalized using logarithmic transformations. At baseline, group differences were identified via chi-square test (comorbidities and drug therapy prevalence) or unpaired Student’s t-test (continuous variables). The effects of the interventions were assessed using a mixed two-way ANOVA (Statsoft, Statistic for Windows 4.3, Oklahoma, USA), the groups being the between factor, and the study phase (baseline and 12 weeks) being the within factor. Newman-Keuls post-hoc tests were used when necessary. P<0.05 was considered significant, and data were presented as mean ± SD.

## Results

Patients flowchart is shown in Figure 2. Eighty-four patients were screened, but 35 were excluded for not meeting the eligibility criteria (n=7) or declining participation (n=28, not available to perform training sessions). The remaining 49 patients were randomly allocated in the CG (n=24) and the WTG (n=25). Fourteen patients withdrew due to circumstances unrelated to the the study. Thus, the final sample was composed of 35 patients (CG, n=16; WTG, n=19).

**Figure 2:** Participants flowchart. IC: Intermittent claudication, CG: Control group, WTG: Walking training group

These groups had similar initial characteristics regarding age, obesity level, clinic BP levels, disease limitations, comorbidities, and medication use (Table 1).

**Table 1:** Characteristics of the patients allocated in the control (CG) and the walking (WTG) training groups.

	CG (n = 16)	WTG (n = 19)	p value
Age (years)	62 ± 7	63 ± 7	0.64
Body mass index (kg/m^2^)	25.7 ± 3.9	26.1 ± 3.1	0.76
Ankle brachial index	0.60 ± 0.12	0.62 ± 0.14	0.61
Claudication onset distance (m)	319 ± 152	277 ± 164	0.45
Total walking distance (m)	759 ± 305	624 ± 255	0.16
Clinic systolic BP (mmHg)	136 ± 19	133 ± 14	0.60
Clinic diastolic BP (mmHg)	79 ± 10	77 ± 9	0.53
**Comorbidities**			
Obesity (%)	12.5	10.5	0.55
Hypertension (%)	81.3	84.2	0.89
Diabetes Mellitus (%)	25.0	21.1	0.61
Dyslipidemia (%)	100.0	89.5	0.17
Current Smokers (%)	37.5	26.3	0.38
Heart Disease/Stroke (%)	18.8	21.1	0.80
**Drug therapy**			
Aspirin (%)	93.8	100.0	0.28
Statin (%)	62.5	78.9	0.83
Angiotensin-converting enzyme inhibitor (%)	43.8	68.4	0.20
Diuretics (%)	25.0	47.4	0.17
Calcium channel blocker (%)	18.8	21.1	0.86
Oral hypoglycemic (%)	18.8	15.8	0.69
**Number of antihypertensive**			
Monotherapy	50.0		

*Data are shown as mean* ± *SD or percentage (%). BP: Blood pressure. Continuous variable* – *unpaired Student’s t-test. Categorical variable* – *chi-square test*.

Ambulatory BP levels were similar between WTG and CG at baseline, and neither group presented any significant change in 24-hour, awake and asleep BPs after the 12 weeks of intervention (Table 2).

**Table 2:** Ambulatory blood pressure levels measured at baseline and after the 12-week intervention period for the walking training (WTG) and the control (CG) groups

	CG (n = 16)		WTG (n = 19)		P group	P study phase	P interaction
	Baseline	12 weeks	Baseline	12 weeks			
**24h**							
Systolic BP (mmHg)	130 ± 14	132 ± 15	128 ± 14	126 ± 11	0.51	0.74	0.21
Diastolic BP (mmHg)	78 ± 7	80 ± 7	78 ± 12	76 ± 10	0.44	0.42	0.16
Mean BP (mmHg)	96 ± 9	98 ± 8	94 ± 9	93 ± 9	0.32	0.60	0.14
**Awake**							
Systolic BP (mmHg)	135 ± 14	137 ± 16	130 ± 14	129 ± 12	0.16	0.74	0.44
Diastolic BP (mmHg)	83 ± 7	84 ± 7	80 ± 12	79 ± 11	0.16	0.41	0.35
Mean BP (mmHg)	101 ± 9	103 ± 9	96 ± 10	95 ± 10	0.08	0.60	0.25
**Asleep**							
Systolic BP (mmHg)	119 ± 16	121 ± 16	124 ± 16	122 ± 12	0.50	0.85	0.51
Diastolic BP (mmHg)	69 ± 9	71 ± 8	73 ± 9	71 ± 11	0.61	0.80	0.32
Mean BP (mmHg)	87 ± 11	89 ± 11	89 ± 9	89 ± 9	0.63	0.82	0.33

*Data are shown as mean* ± *standard deviation. BP: Blood pressure. Mixed two-way ANOVA, with the group being the between main factor and the study phase being the within main factor*.

BP variability indices assessed at baseline were similar between WTG and CG. There was a significant interaction between group and study phase for systolic and mean BP variability indices (all p<0,05), showing a reduction in SD_24_, SD_dn_, and AVR_24_ of systolic and mean BP in the WTG (Table 3, Figure 3). Neither group had any significant change in the indices of diastolic BP variability.

**Table 3:** Ambulatory blood pressure variability indices assessed at baseline and after the 12-week intervention period for the walking training (WTG) and the control (CG) groups

	CG (n = 16)		WTG (n = 19)		P value group	P value study phase	P value interaction
	Baseline	12 weeks	Baseline	12 weeks			
**SD_24_**							
Systolic BP (mmHg)	14.6 ± 3.0	15.5 ± 3.9	13.3 ± 2.8	11.8 ± 2.3*#	0.01	0.65	0.04
Diastolic BP (mmHg)	10.9 ± 1.8	11.2 ± 1.7	9.7 ± 2.3	10.0 ± 2.5	0.06	0.49	0.68
Mean BP (mmHg)	12.0 ± 2.6	13.0 ± 3.0	11.0 ± 1.7	10.4 ± 1.9#	0.01	0.71	0.04
**SD_dn_**							
Systolic BP (mmHg)	12.2 ± 2.4	12.7 ± 3.0	12.1 ± 2.4	10.7 ± 2.5*#	0.18	0.27	0.03
Diastolic BP (mmHg)	8.7 ± 1.3	9.0 ± 1.6	9.0 ± 1.8	8.9 ± 2.2	0.98	0.95	0.48
Mean BP (mmHg)	10.0 ± 2.1	10.7 ± 2.2	10.1 ± 1.6	9.1 ± 1.7*#	0.23	0.82	0.01
**ARV_24_**							
Systolic BP (mmHg)	9.4 ± 2.1	10.7 ± 2.4*	9.4 ± 2.3	8.8 ± 2.2#	0.18	0.28	0.02
Diastolic BP (mmHg)	6.9 ± 1.8	7.3 ± 1.8	7.3 ± 2.3	7.2 ± 1.6	0.75	0.67	0.54
Mean BP (mmHg)	8.1 ± 1.9	8.6 ± 1.7	8.0 ± 1.7	7.2 ± 1.5*#	0.15	0.88	0.01

Values are shown as mean ± standard deviation. SD24 = 24-hour weighted standard deviation; SDdn: awake and asleep weighted standard deviation; ARV: average real variability. Mixed two-way ANOVA, with the group being the between main factor and the study phase being the within main factor.

* Different from baseline (P<0.05);

# Different from CG (P<0.05)

**Figure 3:** Absolute change (Δ) of ambulatory blood pressure variability for the control group (white bars) and walking training group (black bars). BP: blood pressure; SD_24_: standard deviation over 24 hours weighted for the time interval between consecutive readings; SD_dn_: the average of the daytime and nighttime SDs weighted for the duration of the daytime and nighttime interval; ARV_24_: the average real variability weighted for the time interval between consecutive readings in 24-hour ambulatory BP recordings. *p<0.05 vs control group.

## Discussion

The main finding of this study was that 12 weeks of WT decreased systolic and mean BP variability indices without changing ambulatory BP levels.

In the present study, 12 weeks of WT did not alter ambulatory BP in patients with PAD, which contrasts with studies with normotensive subjects and hypertensive patients^19^ that have consistently reported decreases around 3 mmHg for systolic and diastolic ambulatory BP after aerobic training. However, 12 weeks of resistance training have also not changed ambulatory BP in patients with PAD.^9^ Thus, it has been hypothesized that the frequent episodes of ischemia during daily activities in patients with PAD produce claudication pain, oxidative stress and metabolic accumulation, increasing sympathetic nerve activity and, consequently, blunting any possible hypotensive effect of exercise training on ambulatory BP levels.^20^ Another potential explanation, however, can be the too short duration of the training program, since a previous study^21^ conducted with elderly hypertensive patients showed no change in ambulatory BP levels after 6 months of training, but a significant reduction after 12 months.

Despite the absence of change in ambulatory BP levels, reductions in ambulatory systolic and mean BP variabilities were observed for all variability indices: SD_24_, SD_dn_ and ARV_24_. These results are in accordance with a previous study with resistance training in symptomatic PAD patients.^9^ In addition, this result is coherent with the concept that changes in autonomic control precede alterations in BP levels, since BP variability mainly reflects autonomic control of BP.^22^^,^^23^ Additionally, these results are also in accordance with our previous clinic findings of improvements in cardiac autonomic modulation and baroreflex sensitivity, all markers of autonomic control, after WT in patients with PAD.^7^ The absence of changes in diastolic ambulatory BP variability is also coherent with the absence of effects of walking training on calf vascular resistance, as previously described.^7^

Even without any changes in ambulatory BP levels, the decrease in ambulatory BP variability obtained with WT may have relevant clinical implications. BP variability has been associated with the presence and progression of subclinical organ damage as well as the incidence of hard endpoints such as cardiovascular events^10^, leading to a worse cardiovascular prognosis.^8^ Thus, the decrease induced by WT may have favorable impact on the cardiovascular risk of patients with PAD, reinforcing the recommendation of WT for these patients.

This study has some limitations that should be acknowledged. It was conducted only with men, and training-induced adaptations may differ between genders.^24^^,^^25^ Thus, future studies should investigate the impact of WT on ambulatory BP and its variability also in women, especially the elderly, who may experience greater cardiovascular risk than men.^24^ The current study also only examined patients with claudication symptoms, and further studies should examine the effects of WT in other groups of patients, such as those who are asymptomatic (stage 1) and may also present a decrease in ambulatory BP levels after WT. Finally, the training program lasted 12 weeks, a length that improves functional capacity and clinic cardiovascular parameters in these patients,^7^ but a longer training period may be necessary to decrease ambulatory BP levels.

## Conclusion

In conclusion, 12 weeks of WT decreases ambulatory BP variability in men with symptomatic PAD.
